# Occupational sunscreen use among US Hispanic outdoor workers

**DOI:** 10.1186/s13104-015-1558-1

**Published:** 2015-10-17

**Authors:** Ashley K. Day, Jerod L. Stapleton, Ana M. Natale-Pereira, James S. Goydos, Elliot J. Coups

**Affiliations:** Rutgers Cancer Institute of New Jersey, Rutgers, The State University of New Jersey, 195 Little Albany Street, New Brunswick, NJ 08901 USA; School of Psychology, University of Adelaide, North Tce Campus, Adelaide, SA 5005 Australia; Department of Medicine, Rutgers Robert Wood Johnson Medical School, 125 Paterson Street, New Brunswick, NJ 08901 USA; Department of Health Education and Behavioral Science, Rutgers School of Public Health, 683 Hoes Lane West, Piscataway, NJ 08854 USA; Department of Medicine, Rutgers New Jersey Medical School, 185 South Orange Avenue, Newark, NJ 07103 USA; Department of Surgery, Rutgers Robert Wood Johnson Medical School, 125 Paterson Street, New Brunswick, NJ 08901 USA

**Keywords:** Sun protection, Skin cancer, Hispanic, Outdoor workers, UVR

## Abstract

**Background:**

Occupational ultraviolet radiation (UVR) exposure is a risk factor for skin cancer, and Hispanic individuals are over-represented in a number of outdoor occupations (e.g., farming, landscaping). This study examined predictors of occupational sunscreen use in a group of US Hispanic adults who work outdoors.

**Results:**

A population-based sample of outdoor workers (*n* = 149, 85 % male) completed survey measures regarding their demographics, melanoma risk, perceived skin cancer risk, skin cancer knowledge, and their occupational sunscreen use. Sixty-nine percent of the sample reported never or rarely wearing sunscreen while working outdoors. Being female (*p* *=* .02), having a higher level of education (*p* *=* .03), and residing at a higher latitude (*p* *=* .04) were associated with more frequent sunscreen use.

**Conclusions:**

This study highlights the importance of interventions to promote sun protection behaviors among US Hispanic outdoor workers, and identifies potential intervention targets.

## Background

Hispanic individuals in the United States (US) are an important population to target for skin cancer prevention efforts. The incidence of melanoma, the most deadly type of skin cancer, increased among US Hispanics by over 11 % from 1992 to 2011 [1]. Compared to non-Hispanic white individuals, Hispanics are more likely to be diagnosed with later stage and larger melanomas, which increases disease morbidity and mortality [2, 3]. Occupational exposure to ultraviolet radiation (UVR) is a risk factor for skin cancer [4, 5], and Hispanic individuals are over-represented in a number of outdoor occupations. Approximately 25 % of the 2012 US Hispanic workforce were employed in industries that involve significant amounts of occupational UVR exposure, including landscaping, farming, and construction [6]. Thus, it is important to understand the sun protection behaviors of US Hispanic outdoor workers.

A recent systematic review of the US Hispanic skin cancer prevention literature identified only a small number (*n* = 12) of published studies [7]. Regular sunscreen use among samples was low, and sun protection behavior in general (e.g., seeking shade, wearing protective clothing and hats) was “largely suboptimal” [7, p. 584]. Few studies have focused specifically on the sun protection behaviors or skin cancer risk perceptions of US Hispanic outdoor workers. In a 2005 study of 326 California farm workers, 97 % of participants reported never wearing sunscreen during summer months, 35 % reported having no knowledge about skin cancer, and 63 % either did not know if they were at risk, or felt they were not at risk, for developing skin cancer [8]. A 2014 study of Hispanic migrant farm workers in North Carolina reported that 91 % of workers never or rarely used sunscreen and approximately 80 % had low perception and knowledge of skin cancer risks [9]. Individuals with fairer skin reported greater use of sun protection. Given the elevated skin cancer risk associated with occupational UVR exposure, there is a need to better understand factors associated with Hispanic outdoor workers’ engagement in sun protection behaviors.

The present study explored the prevalence and correlates of occupational sunscreen use among Hispanic outdoor workers. Sunscreen use is an important sun protection behavior because it can reduce the risk of premature skin aging (photoaging), squamous cell carcinoma skin cancer, and potentially melanoma [10, 11]. Further, workplace uniform or clothing restrictions can limit the use of other sun protection strategies such as wearing long pants or a long-sleeve shirt [12], making sunscreen a particularly important sun protection approach in this group. Potential correlates of occupational sunscreen use were guided by prior research findings [9, 13] and included sociodemographic characteristics (gender, age, level of education, linguistic acculturation, and latitude of residence), objective risk for melanoma, perceived skin cancer risk, skin cancer knowledge, and the amount of time spent working outside. We anticipated that participants would report low skin cancer knowledge and perceived skin cancer risk, and report infrequent occupational sunscreen use. With respect to correlates, we hypothesized that occupational sunscreen use would be more common among women, individuals with a higher level of education, those with greater objective and perceived skin cancer risk, and those with higher skin cancer knowledge. By identifying correlates of occupational sunscreen use among Hispanic outdoor workers, the current study provides valuable insight on the focus and content of future skin cancer prevention interventions for this understudied and growing population.

## Methods

The data were drawn from a larger survey study of skin cancer risk, prevention, and surveillance behaviors among 788 Hispanic adults. Prior papers have focused on behaviors among the full study sample [13, 14]. The present study focuses on the participants who reported working outdoors, and the analyses reported in this paper have not been previously published.

### Procedure and participants

A detailed description of the study procedures is available elsewhere [13]. In brief, on receiving ethics approval from the Rutgers Health Sciences Institutional Review Board, participants were recruited from KnowledgePanel Latino^SM^, which is a nationally representative web panel of US Hispanic adults (administered by GfK Custom Research). Individuals are recruited to the panel using random-digit dialing and address-based sampling, and are provided with a cost-free laptop and Internet access, if necessary. Panel members residing in five southern and western states (Arizona, California, Florida, New Mexico, or Texas) were selected at random and invited via email to take part in an online survey in either English or Spanish. Informed consent was obtained from all individual participants included in the study. For the current study, we focused on the 149 individuals (from the full sample of 788) who reported working in a job that required them to be outdoors in the sun.

### Measures

#### Sociodemographic factors

Participants indicated their gender, age, level of education, linguistic acculturation, Hispanic heritage, and state of residence. Latitude of residence (i.e., degrees north of the equator) was also calculated for participants, based on their home address.

#### Melanoma risk factors

Objective risk for melanoma was assessed using eight questions regarding melanoma risk factors (e.g., naturally red or blonde hair, history of severe sunburn with blisters, presence of freckles and moles, fair untanned skin). The total number of melanoma risk factors (0–8) was calculated for each participant.

#### Perceived skin cancer risk

Two items adapted from prior research measured perceptions of skin cancer risk [e.g., “If I don’t protect my skin from the sun, I feel that my chances of getting skin cancer in my lifetime are high”; 15]. Participants answered using a 5-point response scale from *strongly disagree* to *strongly agree* and responses were averaged for the two items (Cronbach’s alpha = .93).

#### Skin cancer knowledge

Skin cancer knowledge was assessed using eight true–false items adapted from prior research [16, 17]. The total number of correct items was calculated for each participant.

#### Outdoor work

Participants reported the average number of hours per week that they spend working outside in the sun. An open-ended question asked participants to report the type of work they do outside. Participants also indicated which areas of the body (i.e., face and neck, arms, legs, and trunk of body) are covered by clothing or a hat when working outside in the sun.

#### Occupational sunscreen use

Participants completed a standard survey item (using a 5-point response scale from *never* to *always*) regarding the frequency with which they use sunscreen when engaged in outdoor work in the sun. Due to the distribution of responses (see below), the variable was recoded to represent infrequent (*never* or *rarely*) versus regular sunscreen use (*sometimes, often,* or *always*).

### Statistical analysis

Descriptive statistics were used to describe the sample. Univariable and multivariable logistic regression analyses were conducted to examine correlates of occupational sunscreen use (coded as never/rarely vs. at least sometimes). As described elsewhere [13], the statistical analyses were weighted to adjust for numerous factors, including the probability of panel selection and potential post-stratification non-response and non-coverage biases. A cut-off of *p* < 0.05 was used to determine statistical significance for all analyses.

## Results

### Sample characteristics

Descriptive statistics for the study variables are shown in Table 1. Participants were 85.2 % male and had a mean age of 38.38 years (*SD* = 12.13; range = 19–73). The most common types of outdoor work reported by participants were construction (24.9 %) and landscaping (14.3 %). Predominantly, participants lived in California (52.0 %) or Texas (28.5 %). Participants were primarily of Mexican heritage (73.8 %) with the second most prevalent heritage being Central American (9.9 %). The most commonly reported objective melanoma risk factors were: ever had a severe sunburn with blisters (44.4 %); would get a severe or moderate sunburn if exposed to midday sun without protection (35.9 %); have at least one mole larger than a pencil eraser (approximately 0.6 cm) (35.5 %); and have very fair or fair untanned skin (30.1 %). Nearly half of participants (46.3 %) were Spanish language-acculturated; 38.8 % were considered to be bicultural; and 14.8 % were English-acculturated.

**Table 1:** Descriptive statistics for the study variables

Variable	Weighted % (unweighted no.)	Weighted mean (SD)
Female	14.8 (26)	
Age		38.38 (12.13)
Level of education		
Less than high school	38.0 (51)	
High school	26.6 (43)	
At least some college	35.4 (55)	
Linguistic acculturation		
Spanish-acculturated	46.3 (62)	
Bicultural	38.8 (60)	
English-acculturated	14.8 (25)	
Hispanic heritage		
Mexican	73.8 (110)	
Central American	9.9 (12)	
South American	4.6 (5)	
Cuban	3.8 (6)	
Puerto Rican	2.4 (5)	
Other	5.5 (9)	
State of residence		
Arizona	7.0 (11)	
California	52.0 (80)	
Florida	11.6 (17)	
New Mexico	0.9 (2)	
Texas	28.5 (39)	
Latitude of residence (degrees)		32.54 (3.74)
Objective melanoma risk factors (no.)		1.83 (1.24)
0	12.7 (19)	
1	28.7 (38)	
2	36.6 (49)	
3	12.2 (24)	
≥4	9.9 (19)	
Perceived skin cancer risk		3.58 (1.12)
Skin cancer knowledge		3.92 (1.92)
Hours working outdoors (per week)		25.59 (16.89)
Outdoor occupation		
Construction	24.9 (33)	
Landscaping	14.3 (20)	
Maintenance	9.0 (12)	
Transportation	8.4 (16)	
Farming	4.9 (10)	
Other/unspecified	38.4 (58)	
Occupational sunscreen use		
Never	42.9 (68)	
Rarely	26.1 (37)	
Sometimes	12.0 (23)	
Often	6.9 (10)	
Always	12.1 (11)	
Areas of body covered by clothing when working outside in sun		
Face and neck	18.6 (26)	
Arms	41.1 (57)	
Legs	82.8 (121)	
Trunk of body	93.7 (140)	

### Occupational sun protection

Participants reported working, on average, between 1 and 65 h outdoors in the sun each week (*M* = 25.59 h, *SD* = 16.89). Nearly half of the sample (42.9 %) reported never wearing sunscreen when working outside in the sun, 26.1 % reported doing so rarely, 12.0 % indicated they sometimes wear sunscreen, 6.9 % stated they often wear sunscreen, and 12.1 % of the sample reported that they always wear sunscreen when working outdoors. Participants also reported low levels of sun protective hat or clothing use, particularly protection of their face, neck, and arms (see Table 1).

### Logistic regression analyses examining correlates of occupational sunscreen use

The results of the univariable and multivariable logistic regression analyses examining correlates of occupational sunscreen use are shown in Table 2. Univariable analyses indicated that individuals were more likely to report regular occupational sunscreen use if they were female, had some college education compared to less than high school education, and lived in an area further north of the equator. In the multivariable analysis, occupational sunscreen use was not associated with age, acculturation, number of objective melanoma risk factors, perceived skin cancer risk, skin cancer knowledge, or average hours spent working outdoors. Female gender and college education remained significant in the multivariate analysis.

**Table 2:** Correlates of regular occupational sunscreen use

	Bivariate logistic regression		Multivariate logistic regression (*n* = 142)	
	OR (95 % CI)	*p*	AOR (95 % CI)	*p*
Gender		.02		.02
Male	1 [Reference]		1 [Reference]	
Female	2.86 (1.20, 6.80)		3.54 (1.19, 10.59)	
Age	1.00 (0.98, 1.03)	.80	1.01 (0.97, 1.04)	.70
Level of education		.03		.05
Less than high school	1[ Reference]		1 [Reference]	
High school graduate	1.71 (0.71, 4.12)		2.51 (0.85, 7.38)	
At least some college	2.87 (1.30, 6.35)		3.79 (1.29, 11.12)	
Linguistic acculturation		.54		.84
Spanish-acculturated	1 [Reference]		1 [Reference]	
Bicultural	1.50 (0.73, 3.05)		1.24 (0.41, 3.71)	
English-acculturated	1.29 (0.48, 3.43)		0.89 (0.19, 4.21)	
Latitude of residence (degrees)	1.11 (1.01, 1.21)	.04	1.10 (0.99, 1.23)	.08
Objective melanoma risk factors (no.)	1.08 (0.83, 1.40)	.56	0.99 (0.70, 1.39)	.94
Perceived skin cancer risk	1.31 (0.96, 1.78)	.09	1.38 (0.94, 2.02)	.10
Skin cancer knowledge	1.16 (0.97, 1.39)	.10	1.06 (0.83, 1.37)	.64
Hours working outdoors (per week)	1.00 (0.98, 1.02)	.83	1.01 (0.99, 1.04)	.35

Sunscreen use was coded as never/rarely vs. regular use (i.e., at least sometimes)

## Conclusions

The present study examined the prevalence and correlates of occupational sunscreen use among US Hispanic outdoor workers. Consistent with previous research involving US Hispanic outdoor workers [8, 9], reported rates of occupational sunscreen use were low. The majority of the sample (69 %) reported never or rarely wearing sunscreen while working outdoors in the sun, and only 12.1 % stated that they always used sunscreen when working outdoors. Information about sunscreen benefits should be incorporated into skin cancer prevention programs targeting this population group.

As hypothesized, participants were more likely to report regular occupational sunscreen use if they were female, lived in an area further north of the equator, and had some college education compared to less than high school education. Objective risk for melanoma and perceived skin cancer risk were not associated with occupational sunscreen use in the present sample. A review of the literature showed that sunscreen use among general adult populations is associated with having a greater number of skin cancer risk factors or reported “sun-sensitivity” [18]. Hispanics typically have fewer objective melanoma risk factors (e.g., fair untanned skin, fair hair, blue or green eyes) than non-Hispanic white individuals. The majority of participants in the current study had two or fewer objective melanoma risk factors, suggesting the presence of floor effects, which may account for the non-significant result.

Perceived skin cancer risk scores were higher in the present sample than previous work [8, 9], indicating that the present participants were aware that sun exposure increased their risk of skin cancer. Despite this awareness, skin cancer knowledge levels were low. This is consistent with prior research among US Hispanic outdoor workers [8, 9], as well as US Hispanics in general [19–21]. Low skin cancer knowledge may contribute to delayed diagnosis seeking of Hispanics with skin cancers. This may in turn increase their likelihood of being diagnosed with later stage, larger skin cancers that are associated with poorer disease morbidity and mortality [2, 3]. Thus, educational information about skin cancer prevention methods and identifying skin cancer should be incorporated into skin cancer prevention programs targeting this population group.

Barriers to sun protection have been associated with lower levels of sun protection behaviors among Hispanic and general US adult populations [14, 18], including among outdoor workers [22]. Perceived sunscreen barriers include taking too long to apply, causing discomfort or irritation, and financial expense [23]. Future research should consider the role of perceived barriers of occupational sunscreen use among US Hispanic outdoor workers, and consider interventions that address relevant barriers. Recent research found that sunscreen was less available in Hispanic compared to non-Hispanic neighborhoods in Chicago, and that product choice was more limited [24]. Environmental factors such as product availability should be considered when planning interventions.

The present research is the first known study to explore predictors of occupational sunscreen use among a multi-state US Hispanic participant group who work in a variety of outdoor roles. A probability-based sampling approach was utilized, and both English- and Spanish- language questionnaires were available to participants. However, the findings of this study are subject to some limitations. As this study is cross-sectional in design, inferences cannot be drawn regarding the causal direction of the observed associations. This study included participants from five US states and the extent to which the results can be extrapolated to US Hispanic outdoor workers residing in other states is unclear.

The present study identified the prevalence and correlates of occupational sunscreen use among US Hispanic adults who work outdoors. Future research should consider the role of barriers to occupational sunscreen use in this population group. These results highlight the importance of interventions to promote sun protection behaviors among US Hispanic outdoor workers.

## Abbreviations

US: United States
UVR: ultraviolet radiation
